# Longitudinal changes in medial meniscus extrusion and clinical outcomes following pullout repair for medial meniscus posterior root tears: a 3-year evaluation

**DOI:** 10.1007/s00590-024-03889-8

**Published:** 2024-03-22

**Authors:** Koki Kawada, Takayuki Furumatsu, Yusuke Yokoyama, Naohiro Higashihara, Masanori Tamura, Toshifumi Ozaki

**Affiliations:** Department of Orthopaedic Surgery, Okayama University Graduate School of Medicine, Dentistry, and Pharmaceutical Sciences, 2-5-1 Shikata-cho, Kita-ku, Okayama, 700-8558 Japan

**Keywords:** Meniscus, Posterior root tear, Meniscus extrusion, Osteoarthritis, Clinical score

## Abstract

**Purpose:**

We aimed to evaluate the longitudinal changes in medial meniscus extrusion (MME) and clinical scores at multiple time points up to 3 years after pullout repair for medial meniscus posterior root tears (MMPRTs).

**Methods:**

This retrospective case series study included 64 patients who underwent pullout repair for MMPRTs and four MRI evaluations (preoperatively and at 3 months, 1 year, and 3 years postoperatively). MME was measured during the same time points. Clinical scores were assessed four times: preoperatively and at 1, 2, and 3 years postoperatively. Additionally, a multivariate analysis was performed on the change in MME (ΔMME) from the preoperative measurement point to 3 years postoperatively.

**Results:**

The ΔMME per month from the preoperative measurement point to 3 months postoperatively, from 3 months to 1 year postoperatively, and from 1 to 3 years postoperatively were 0.30, 0.05, and 0.01 mm/month, respectively. All clinical scores significantly improved 3 years postoperatively (*p* < 0.001). In a multiple regression analysis for ΔMME from the preoperative measurement point to 3 years postoperatively, sex significantly affected the outcome (*p* = 0.039).

**Conclusion:**

Following pullout repair for MMPRTs with well-aligned lower extremities, although MME progression could not be entirely prevented, the rate of progression decreased over time, and clinical scores improved. In particular, MME progressed markedly during the first 3 months postoperatively. Additionally, sex had a significant influence on MME progression, suggesting that males may be able to expand the indications of pullout repair for MMPRTs.

## Introduction

Medial meniscus (MM) posterior root (MMPR) tears (MMPRTs) have received increasing attention in recent years and are gradually becoming recognized and diagnosed [1]. The long-term results of conservative treatment for MMPRTs are poor, with a reported failure rate of 95% at a minimum of 10 years of follow-up and 64% of cases requiring surgery for total knee arthroplasty [2]. In addition, MMPR repair has been reported to yield good results compared with conservative treatments and meniscectomy [3–5]. Consequently, MMPR repair is being performed more frequently. However, MMPR repair does not completely prevent the progression of osteoarthritic changes [6, 7]. Moreover, it has been reported that there is a correlation between the MM extrusion (MME) progression and the narrowing of the medial joint space [6]. Therefore, a long-term evaluation of MME after MMPR repair is required.

MME progression following pullout repair for MMPRTs remains controversial. Several reports have shown that MME continues progressing postoperatively to varying degrees. Kynch et al. [8] reported an MME progression of 0.9 mm at an average of 6 months following pullout repair. Additionally, Moon et al. [9] showed that MME progressed 1.0 mm on average at 1 year after the procedure. These reports also indicated that MME progresses relatively early in the postoperative period. Conversely, Chung et al. [10] reported an average MME progression of 0.3 mm at 6 years after pullout repair. MME progression after pullout repair for MMPRTs has been reported to be influenced by various factors such as patient characteristics, surgical technique, and rehabilitation. Therefore, a consensus has not yet been reached on the extent and timing of MME progression [11, 12]; most of the previous reports addressing MME progression after pullout repair for MMPRTs have been evaluated at one point in time or at the last follow-up.

To the best of our knowledge, no prior study has evaluated both MME and clinical scores at multiple time points over a 3-year postoperative period. Therefore, we aimed to evaluate the longitudinal changes in MME (ΔMME) and clinical scores across multiple time points up to 3 years after pullout repair for MMPRTs. We hypothesized that while MME progression following MMPR repair may not be completely preventable, the progression decreases over time, coinciding with an improvement in clinical scores.

## Materials and methods

### Patients

This retrospective case series study was performed following the Declaration of Helsinki and approved by our Institutional Review Board (No. 1857). Written informed consent was obtained from all patients.

Between December 2018 and November 2020, a total of 158 patients underwent pullout repair for isolated MMPRTs at our institution. Among them, 69 patients who underwent four magnetic resonance imaging (MRI) evaluations (preoperatively and at 3 months, 1 year, and 3 years postoperatively) were included in the retrospective survey. Finally, 64 patients were evaluated, excluding one case of anterior cruciate ligament insufficiency and four cases of chronic MMPRTs with an unknown onset time.

The surgical indications of pullout repair for the symptomatic MMPRTs at our institution included varus knee alignment ≤ 5°, a Kellgren–Lawrence (KL) grade of 0–2, and mild cartilage lesions. No patient was excluded from surgery based on age, body mass index (BMI), or activity levels. The surgery was performed by a single experienced surgeon. The time from injury to surgery was determined through detailed interviews regarding painful popping episodes.

### Surgical technique and rehabilitation protocol

**Fig. 1:**
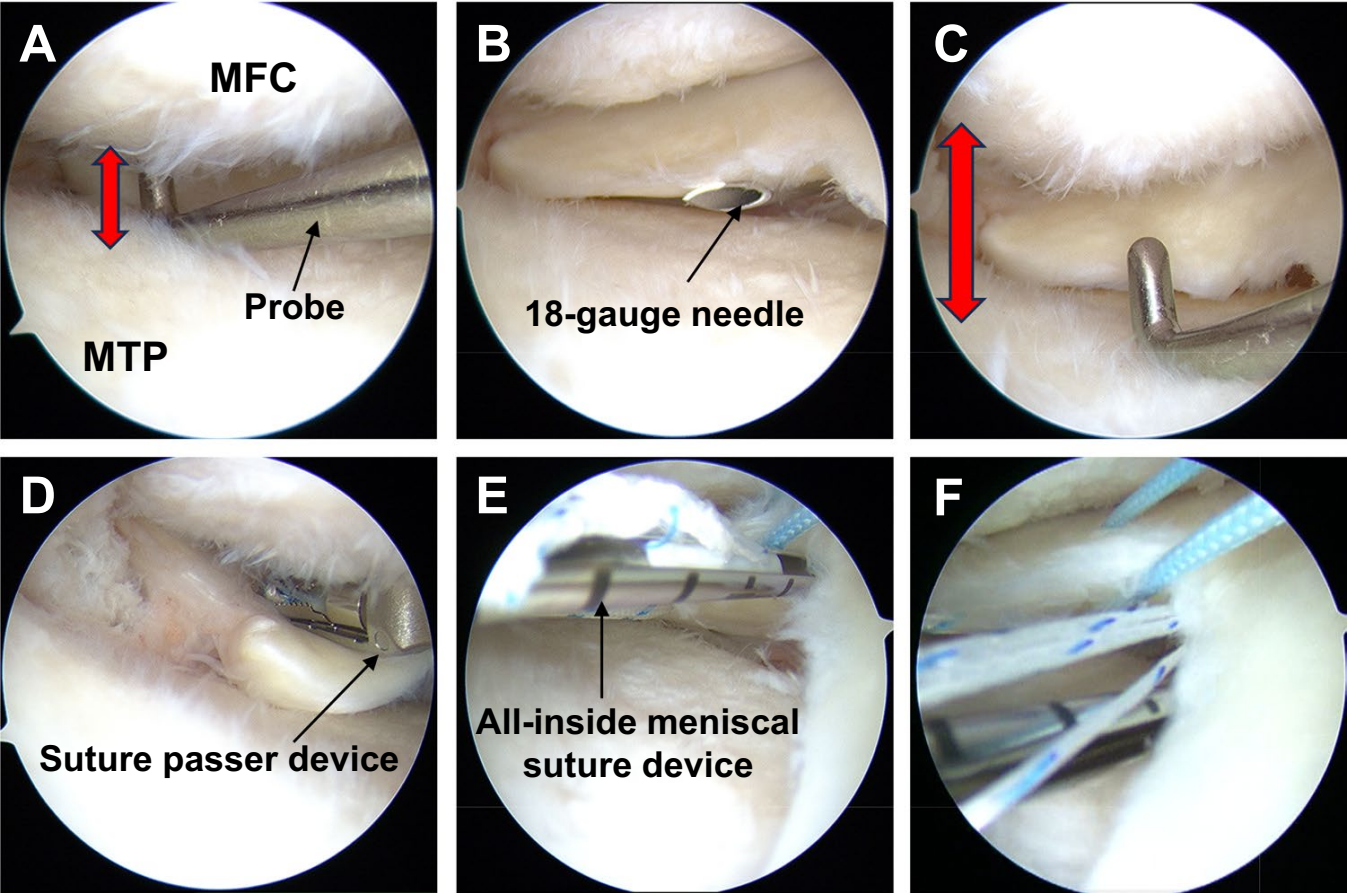
Arthroscopic findings of the surgical technique for diagnosis and suture for MMPRTs. **A** Prior to the outside-in pie-crusting technique, the medial knee compartment appeared narrow and challenging to treat for MMPRTs. **B** Utilizing an outside-in pie-crusting technique with an 18-gauge needle, the posterior one-third of the medial collateral ligament and the posterior oblique ligament were incised. **C** Following the outside-in pie-crusting technique, the medial knee compartment was enlarged, facilitating the diagnosis and treatment of MMPRTs. **D** Two threads were passed 5 and 10 mm from the torn edge of the MMPR using a suture passer device. **E** Additional sutures were performed using an all-inside meniscal suture device, such as the FasT-Fix system. The first needle was inserted into the inferior aspect of the MM posterior horn in a posteromedial direction. **F** The second needle was inserted directly into the articular capsule via the undersurface of the MM. MM, medial meniscus; MMPR, medial meniscus posterior root; MMPRTs, medial meniscus posterior root tears

**Fig. 2:**
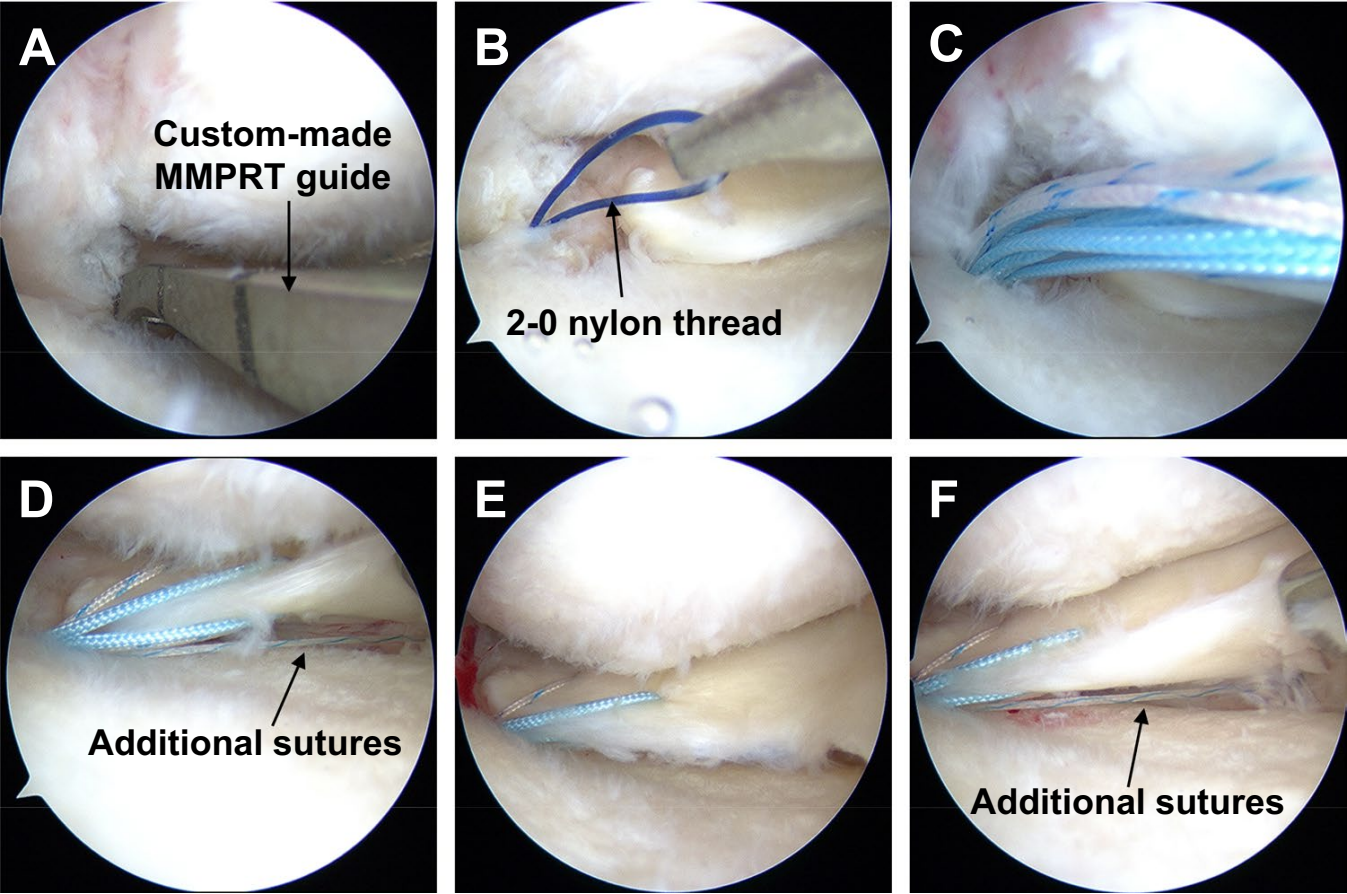
Arthroscopic findings demonstrating the surgical technique of tibial tunnel creation and pullout repair for MMPRTs. **A** A custom-made MMPRT guide was used to insert a guide pin into the anatomic attachment of the MMPR at a 45° angle to the articular plane, followed by overdrilling with a 4.0-mm diameter cannula-type drill to create a tibial tunnel. **B** Using a suture retriever, 2–0 nylon thread was passed through the tibial tunnel. **C** Three sutures were suture relayed together and pulled out into the tibial tunnel. **D** Prior to tibial fixation of sutures, additional sutures were visible in the inferior aspect of the MM. **E** Following tibial fixation of the sutures, additional sutures were concealed under the MM, and the MMPR retracted on the tibial tunnel. **F** After tibial fixation of the sutures, the MM was lifted with a probe, revealing the additional suture. MM, medial meniscus; MMPR, medial meniscus posterior root; MMPRTs, medial meniscus posterior root tears

A pullout repair procedure was used as the surgical technique [13]. The patient was positioned supine with an air tourniquet, and standard anterolateral and anteromedial portals were created. Subsequently, an outside-in pie-crusting technique was performed to enlarge the medial knee compartment (Fig. 1A–C) [14]. The presence of MMPRTs was confirmed and classified according to the system proposed by LaPrade et al. [15]. Two simple stitches or cinch stitches were applied using a suture passer device with No. 2 Ultrabraid or Ultratape (Smith & Nephew, Andover, MA, USA) at the torn edge of the MMPRTs (Fig. 1D). After additional sutures were performed using an all-inside meniscal suture device, such as the FasT-Fix system (Smith & Nephew), the first needle was inserted into the inferior aspect of the MM posterior horn in a posteromedial direction (Fig. 1E), while the second needle was inserted directly into the articular capsule via the undersurface of the MM (Fig. 1F). Subsequently, a custom-made MMPRT guide (Smith & Nephew) [16] was used to insert a guide pin into the anatomic attachment of the MMPR at a 45° angle to the articular plane, followed by overdrilling with a 4.0-mm diameter cannula-type drill to create a tibial tunnel (Fig. 2A). Following this, the three sutures were pulled out into the tibial tunnel (Fig. 2B, C). Tibial fixation of the pullout sutures was performed using an interference screw at 30° of knee flexion with an initial tension of 10–20 N (Fig. 2D–F).

The rehabilitation protocol consisted of immobilizing the knee joint in extension and unloading for 1 week. Subsequently, patients were allowed to increase the knee joint range of motion by 30° and load by 20 kg every other week. The knee joint range of motion was limited to 120° for 2 months postoperatively. Continuously until 3 months postoperatively, patients underwent strength training to strengthen the quadriceps muscles with the support of a physical therapist.

### Magnetic resonance imaging assessments

**Fig. 3:**
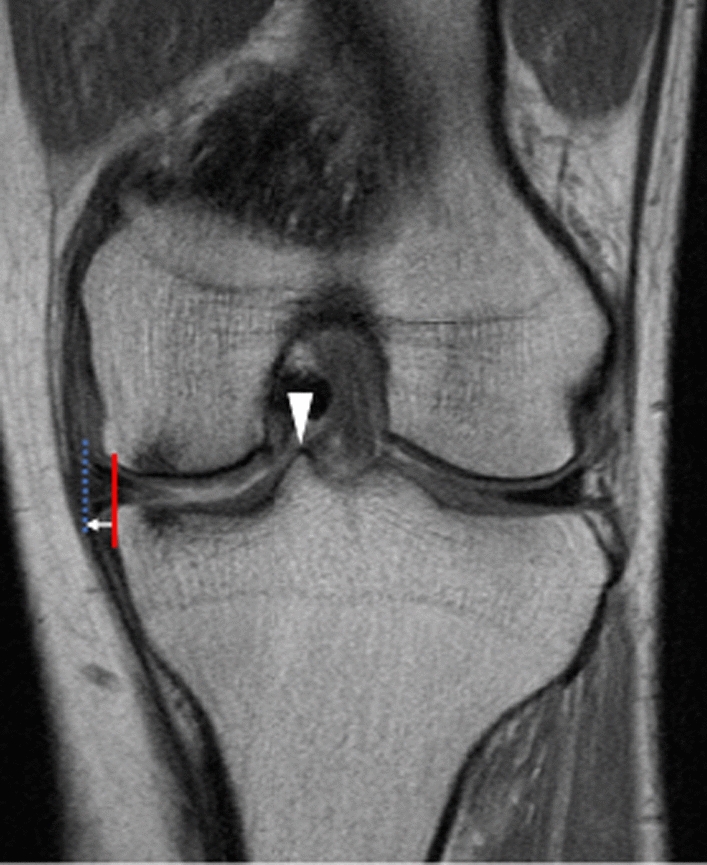
MME measurement. MME (white arrow) was measured as the distance between the medial edge of the MM (blue dotted line) and the tibial plateau excluding the osteophytes (red line) in the slice in which the medial tibial eminence was the highest (white arrowhead) using MRI coronal images. MM, medial meniscus; MME, medial meniscus extrusion; MRI, magnetic resonance imaging

MRI was performed four times: preoperatively and at 3 months, 1 year, and 3 years postoperatively. MME was measured during the same time points. MME was measured as the distance between the medial edge of the MM and the tibial plateau in the slice in which the medial tibial eminence was the highest, using MRI coronal images (Fig. 3).

The healing status of the repaired posterior roots was assessed using MRI at 3 years postoperatively and classified based on a previous study as follows: complete healing (continuity was confirmed in all three MRI views: sagittal, coronal, and axial), partial healing (loss of continuity in any one view), and repeated tears (no continuity in any view) [17].

### Clinical scores

Lysholm scores [18], Tegner activity scores [18], Knee Injury and Osteoarthritis Outcome Score (KOOS) [19], International Knee Documentation Committee (IKDC) scores [20], and pain visual analog scale (VAS) scores [21] were assessed four times: preoperatively and at 1, 2, and 3 years postoperatively. KOOS consists of five subscales: pain, symptoms, activities of daily living (ADL), sport/recreation function (Sport/Rec), and quality of life (QOL).

### Statistical analysis

Measurements were expressed as mean ± standard deviation. Statistical analyses were performed using the EZR software (Saitama Medical Center, Saitama, Japan). Wilcoxon’s signed-rank test was used to compare MME and the clinical scores over time. Patients were divided into two groups based on the median ΔMME from the preoperative measurement point to 3 years postoperatively, and their characteristics, MME, and clinical scores were compared. Furthermore, patient characteristics, ΔMME, and clinical scores were compared between males and females. In addition, a multivariate analysis was performed on ΔMME from the preoperative measurement point to 3 years postoperatively. Furthermore, the Mann–Whitney U test was used to compare ΔMME from the preoperative measurement point to 3 years postoperatively with the healing status of the repaired posterior roots. Statistical significance was set at *p* < 0.05. The inter- and intra-observer reliabilities of MME measurements were determined using the intraclass correlation coefficient.

For the sample size, a post hoc analysis was conducted using MME results preoperatively and at 3 months postoperatively (G*Power, University of Dusseldorf, Dusseldorf, Germany; version 3.1.9.7).

## Results

Sixty-four patients were included in this study (Table 1). The mean age, BMI, and time from injury to surgery were 64.0 ± 7.2 years, 26.1 ± 4.3 kg/m^2^, and 65.3 ± 61.2 days, respectively. The MMPRT classification (1/2/3/4/5) based on the arthroscopic findings at the time of pullout repair was 8/51/0/5/0 (number of patients in each group). A KL grade ≥ 3 was observed in 7 of the 64 cases (10.9%) at 3 years postoperatively. The KL grade progressed by 1 grade in 29 of the 64 cases (45.3%) and by 2 grades in 2 of the 64 cases (3.1%) at 3 years postoperatively.

**Table 1:** Patient characteristics

Characteristic	Value	Range
Patients	64	
Age (years)	64.0 ± 7.2	45–78
Sex, male/female	11/53	
Body mass index (kg/m^2^)	26.1 ± 4.3	17.0–35.5
MMPRT side, left/right	37/27	
Time from injury to surgery (days)	65.3 ± 61.2	9–346
Preoperative varus knee alignment (°)	2.6 ± 1.5	− 2.0 to 5.0
Preoperative posterior tibial slope (°)	9.0 ± 2.9	2.5–15.0
Preoperative KL grade, 0/1/2/3/4	0/40/24/0/0	
Postoperative 3 years KL grade, 0/1/2/3/4	0/14/43/7/0	
MMPRT classification, 1/2/3/4/5	8/51/0/5/0	

Values are presented as the mean ± standard deviation or number

KL, Kellgren–Lawrence; MMPRT, medial meniscus posterior root tear

**Fig. 4:**
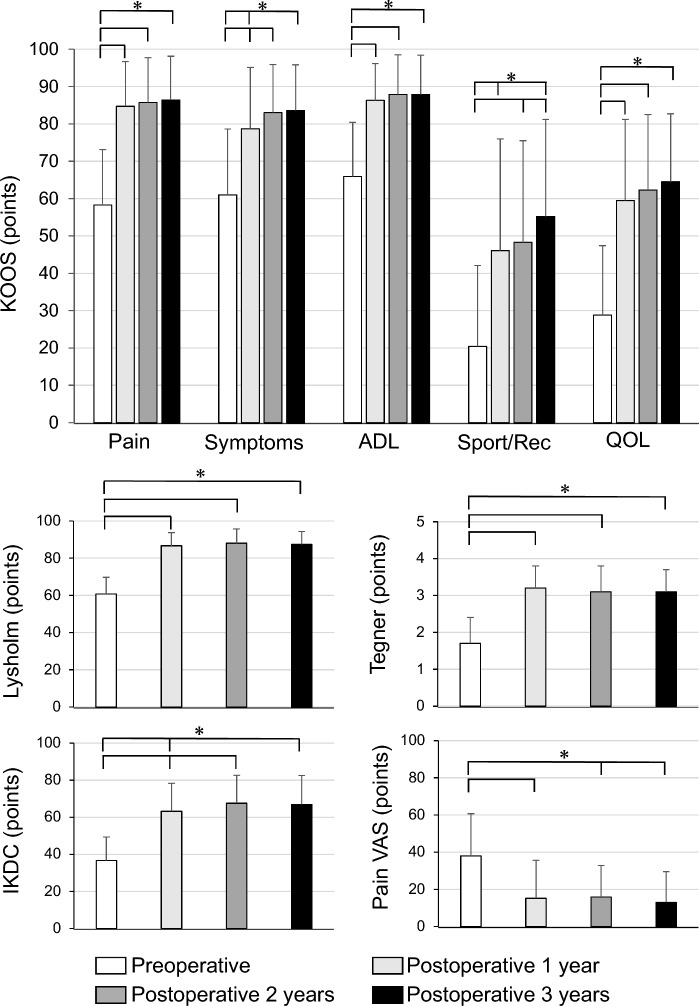
Longitudinal changes in clinical scores. All clinical scores showed significant improvements at 1, 2, and 3 years postoperatively compared with those preoperatively. The KOOS-symptoms (*p* = 0.019 and *p* = 0.003) and IKDC score (*p* = 0.027 and *p* = 0.049) showed significant improvements at 2 and 3 years postoperatively compared with those at 1 year postoperatively. In addition, KOOS-Sport/Rec (*p* = 0.017) and pain VAS score (*p* = 0.033) significantly improved from 2 to 3 years postoperatively. IKDC, International Knee Documentation Committee; KOOS, Knee Injury and Osteoarthritis Outcome Score; Sport/Rec, sport/recreation function; VAS, visual analog scale *p < 0.05

All clinical scores showed significant improvements at 1, 2, and 3 years postoperatively compared with those preoperatively (Fig. 4). KOOS-symptoms (*p* = 0.019 and *p* = 0.003) and the IKDC score (*p* = 0.027 and *p* = 0.049) showed significant improvements at 2 and 3 years postoperatively, respectively, compared with those at 1 year postoperatively. In addition, the KOOS-Sport/Rec (*p* = 0.017) and pain VAS score (*p* = 0.033) significantly improved from 2 to 3 years postoperatively.

As we assessed the healing status of the repaired posterior roots using MRI at 3 years postoperatively, we found complete healing in 60/64 cases (94%) and partial healing in 4/64 cases (6%).

**Fig. 5:**
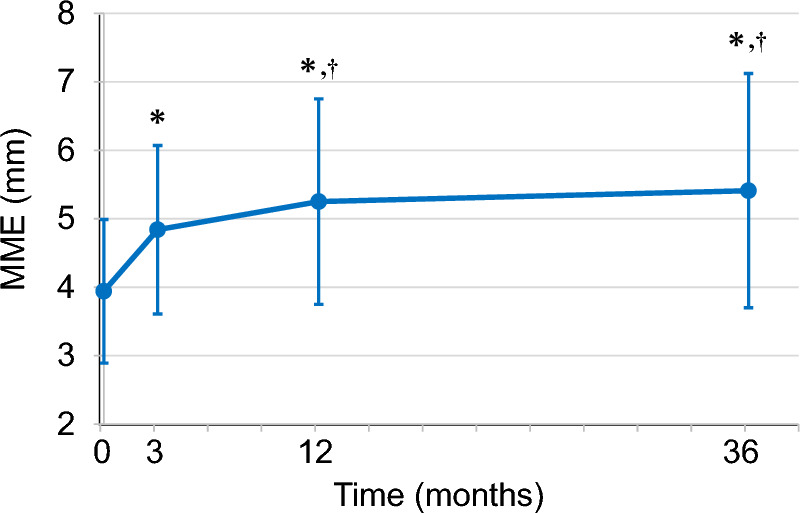
Longitudinal changes in MME. MME preoperatively and at 3 months, 1 year, and 3 years postoperatively was 3.95 ± 1.05, 4.84 ± 1.23, 5.25 ± 1.50, and 5.41 ± 1.71 mm, respectively. MME progression was 0.30 mm/month from the preoperative measurement to 3 months postoperatively, 0.05 mm/month from 3 months to 1 year postoperatively, and 0.01 mm/month from 1 to 3 years postoperatively; thus, indicating that the MME progression rate decreased over time. MME, medial meniscus extrusion. *p < 0.05 (vs. preoperative), ^†^*p* < 0.05 (vs. postoperative 3 months)

MME preoperatively and at 3 months, 1 year, and 3 years postoperatively were 3.95 ± 1.05, 4.84 ± 1.23, 5.25 ± 1.50, and 5.41 ± 1.71 mm, respectively (Fig. 5). MME showed a significant progression at 3 months (*p* < 0.003), 1 year (*p* < 0.001), and 3 years (*p* < 0.001) compared with that measured preoperatively. MME also showed significant progression at 1 year (*p* < 0.001) and 3 years (*p* < 0.001) compared with that measured at 3 months postoperatively. MME did not significantly progress between 1 and 3 years postoperatively (*p* = 0.105).

The ΔMME from the preoperative measurement point to 3 months postoperatively, from 3 months to 1 year postoperatively, and from 1 to 3 years postoperatively were 0.89 ± 0.94, 0.41 ± 0.66, and 0.16 ± 0.58 mm, respectively (Fig. 5). ΔMME per month from the preoperative measurement point to 3 months postoperatively, from 3 months to 1 year postoperatively, and from 1 to 3 years postoperatively were 0.30, 0.05, and 0.01 mm/month, respectively.

The median ΔMME from the preoperative measurement point to 3 years postoperatively was 1.09 mm, dividing the study population into two groups with lower (< 1.09 mm) and higher (≥ 1.09 mm) ΔMME. Furthermore, we observed a significant sex-based difference between the two groups (*p* = 0.043; Table 2). However, the two groups showed no significant differences in either the pre- or postoperative clinical scores.

**Table 2:** Comparison of lower and higher ΔMME from the preoperative measurement point to 3 years postoperatively

	Lower ΔMME group (< 1.09 mm)	Higher ΔMME group (≥ 1.09 mm)	*p*-value
Patients, n	32	32	
Sex, male/female	9/23	2/30	0.043*
Age (years)	64.4 ± 7.2	63.5 ± 7.1	0.488
[Range]	[45–77]	[49–78]	
Body weight (kg)	64.4 ± 10.4	62.5 ± 12.4	0.476
[Range]	[49.0–89.0]	[43.0–85.0]	
Body mass index (kg/m^2^)	26.5 ± 4.1	25.7 ± 4.4	0.550
[Range]	[17.0–34.5]	[19.1–35.5]	
Time from injury to surgery (days)	70.3 ± 56.4	60.4 ± 65.3	0.298
[Range]	[13–238]	[9–346]	
Preoperative varus knee alignment (°)	2.4 ± 1.5	2.9 ± 1.5	0.110
[Range]	[− 2.0 to 5.0]	[− 2.0 to 5.0]	
Preoperative MME (mm)	3.88 ± 1.02	4.03 ± 1.08	0.528
[Range]	[2.08–6.15]	[1.50–6.10]	

Values are presented as means ± standard deviations or numbers. *p*-values were derived using Fisher’s exact or Mann–Whitney U test

MME, medial meniscus extrusion; ΔMME, change in medial meniscus extrusion

*Statistically significant

The male group exhibited a significantly lower ΔMME than the female group (*p* = 0.010; Table 3). There were no significant differences in preoperative clinical scores between sexes. However, Tegner scores (*p* < 0.001) and KOOS-Sport/Rec scores (*p* = 0.041) at 3 years postoperatively were significantly higher in males than in females. In a multiple regression analysis for ΔMME from the preoperative measurement point to 3 years postoperatively, sex was significantly affected (*p* = 0.039; Table 4).

**Table 3:** Comparison of male and female groups

	Male group (n = 11)	Female group (n = 53)	*p*-value
Age (years)	62.5 ± 7.0	64.0 ± 7.2	0.498
Body weight (kg)	71.9 ± 8.3	63.5 ± 11.3	0.006*
Body mass index (kg/m^2^)	26.3 ± 3.3	26.1 ± 4.3	0.689
Time from injury to surgery (days)	85.6 ± 99.2	65.3 ± 61.2	0.715
Preoperative varus knee alignment (°)	2.7 ± 1.6	2.6 ± 1.5	0.907
ΔMME from the preoperative measurement point to 3 years postoperatively (mm)	0.53 ± 0.55	1.45 ± 1.43	0.010*

*p*-values were derived using the Fisher's exact or Mann–Whitney U test

Values are presented as means ± standard deviations

ΔMME, change in medial meniscus extrusion

*Statistically significant

**Table 4:** Multiple regression analysis for ΔMME from the preoperative measurement point to 3 years postoperatively

Variables	β	t-value	*p*-value	95% confidence interval
(Intercept)	2.472	0.903	0.370	– 3.007 to 7.953
Age (years)	− 0.008	− 0.289	0.773	– 0.064 to 0.048
Body weight (kg)	0.029	0.663	0.510	– 0.059 to 0.117
Body mass index (kg/m^2^)	− 0.140	− 1.346	0.184	– 0.349 to 0.068
Sex, male/female	1.309	2.109	0.039*	0.066 to 2.551
Time from injury to surgery (days)	− 0.004	− 1.316	0.193	− 0.010 to 0.002
Preoperative varus knee alignment (°)	0.182	1.524	0.133	− 0.057 to 0.421

Statistical differences were analyzed using multiple regression analysis

β, partial regression coefficient; ΔMME, change in medial meniscus extrusion

*Statistically significant

The ΔMME from the preoperative measurement point to 3 years postoperatively in patients with complete and partial healing were 1.42 ± 1.41 and 1.89 ± 1.69 mm, respectively, with no statistically significant difference (*p* = 0.598).

The intra-observer and inter-observer reliability of MME measurements were 0.935 and 0.948, respectively, using the intraclass correlation coefficient. In a post hoc analysis, the actual power in Wilcoxon signed-rank test to assess MME results preoperatively (3.95 mm) and at 3 months postoperatively (4.84 mm) was 99.8%, with a standard deviation of 1.23, an effect size of 0.72, an α error of 0.05, and a sample size of 64.

## Discussion

The primary finding of this study was that following pullout repair for MMPRTs, although MME progression could not be completely prevented, the rate of MME progression decreased over time whereas the clinical scores improved. In particular, MME progressed markedly during the initial 3 months postoperatively. Additionally, sex had a significant effect on ΔMME.

MME progression is associated with increased joint loading in the medial compartment and progression of osteoarthritis [22]. Furthermore, MME is more advanced in patients with MMPRTs preoperatively than in healthy patients [23]. Therefore, MME is expected to decrease following pullout repair. Although previous reports have indicated that MME progression after repair for MMPRTs cannot be completely prevented, some degree of MME progression is observed [9, 24, 25]. Similarly, we were unable to completely prevent MME progression and measured a progression of 1.45 mm at 3 years following pullout repair for MMPRTs.

Interestingly, the rate of MME progression decreased over time; ΔMME was 0.30 mm/month from the preoperative measurement point to 3 months postoperatively, 0.05 mm/month from 3 months to 1 year postoperatively, and 0.01 mm/month from 1 to 3 years postoperatively. MME progression during the early postoperative stages has been previously reported [8, 9]. However, this does not mean that the MMPR repair failed to restore continuity, as MRI and arthroscopic evaluation did not show a re-tear of the MMPR. In other words, this might indicate that the pullout repair procedure alone may have difficulties reestablishing the original meniscal hoop function. Recently, there have been reports of centralization, meniscal circumferential fiber augmentation, and meniscotibial ligament repair to reestablish the meniscal hoop function [26–28]. However, most of these reports are still short-term and biomechanical studies.

Another critical finding in this present study was that the group with a lower ΔMME had a significantly larger proportion of male patients, compared with the group with a higher ΔMME. Furthermore, in a comparison between males and females, males showed significantly smaller ΔMME values than females. In the multiple regression analysis, sex emerged as a significant factor affecting ΔMME. Although MMPRTs occur approximately four times more frequently in females than in males [29], why the incidence differs by sex or how the sex affects the postoperative clinical outcomes remains to be determined. Kawada et al. [11] reported a correlation between postoperative quadriceps muscle strength and ΔMME. Other possible influences include differences in bone morphology, activity levels, and lifestyle between the sexes. Future investigations need to take these evaluation factors into account when evaluating outcomes across the sexes.

The pullout repair procedure for MMPRTs has been reported to have good clinical scores in the mid- to long term [30]. However, few reports have evaluated the changes in clinical scores over time. Kodama et al. evaluated clinical scores preoperatively and at 3, 6 months, and 1 year postoperatively, and reported improvements in clinical scores over time up to 1 year [31]. In this study, all clinical scores significantly improved from the preoperative measurement to 1, 2, and 3 years postoperatively. In addition, the KOOS-Sport/Rec and pain VAS scores significantly improved from 2 to 3 years postoperatively. This longitudinal evaluation revealed that these scores improved early in the first postoperative year. However, there was a notable improvement in pain, symptoms, and activity levels during the early postoperative period, which then stabilized over time. In contrast, the ability to engage in light sports and recreational activities, such as jumping and running, continued to enhance progressively over the 3 years following surgery. These results are consistent with the early postoperative improvement in pain and subsequent gradual improvement in activity observed in practice with our patients with MMPRTs.

This study underscores two major points of clinical relevance for the future. First, it suggests that the progression of MME may be influenced by surgical techniques and manipulations. MME progression was observed predominantly in the early postoperative period, within 3 months after surgery. In addition, MRI scans conducted 3 years postoperatively confirmed the continuity of the repaired posterior roots, without evidence of re-tears. The observed early postoperative MME progression may have been attributed to damage to the posterior oblique ligament caused by the outside-in pie-crusting technique, as well as to the meniscus tissue due to the pullout repair technique using a suture passer device. Future studies should consider evaluating MME immediately after surgery. Second, the study suggests that males may have a broader indication of pullout repair for MMPRTs. Despite the generally higher body weight of males, their MME progression was found to be smaller than that in females. While factors such as muscle strength and anatomical morphology need to be considered, this finding may imply that, in the future, males could potentially benefit from more selective options for repair surgery.

The strength of this study is that it evaluated MME at multiple time points and confirmed the timing of MME progression. However, this study had some limitations. First, it was retrospective. Second, the sample size was relatively small. Third, the evaluation period was limited to 3 years postoperatively; longer-term assessments may offer a different perspective. Fourth, MRI was only evaluated in the non-weight-bearing position. Fifth, the MME measurements were taken at relatively narrow intervals and limited time points; a more comprehensive evaluation of MME changes over time from additional time points would be beneficial. Finally, there was a potential for selection bias as not all patients were able to undergo four MRI scans.

## Conclusions

Following pullout repair for MMPRTs with well-aligned lower extremities, although complete prevention of MME progression was not achieved, the rate of progression decreased over time and clinical scores improved. In particular, MME progressed markedly during the first 3 months postoperatively. Additionally, sex had a significant influence on MME progression, suggesting that males may be able to expand the indications of pullout repair for MMPRTs.

## Data Availability

The datasets generated and analyzed during the current study are available from the corresponding author on reasonable request.
